# Microcontact Printing of Biomolecules on Various Polymeric Substrates: Limitations and Applicability for Fluorescence Microscopy and Subcellular Micropatterning Assays

**DOI:** 10.1021/acsapm.2c00834

**Published:** 2022-09-06

**Authors:** Roland Hager, Christian Forsich, Jiri Duchoslav, Christoph Burgstaller, David Stifter, Julian Weghuber, Peter Lanzerstorfer

**Affiliations:** † School of Engineering, University of Applied Sciences Upper Austria, 4600 Wels, Austria; ‡ Center for Surface and Nanoanalytics (ZONA), Johannes Kepler University Linz, 4040 Linz, Austria; § Transfercenter für Kunststofftechnik GmbH, 4600 Wels, Austria; ∥ FFoQSI—Austrian Competence Center for Feed and Food Quality, 3430 Tulln, Austria

**Keywords:** subcellular micropatterning, polymeric biointerfaces, protein−protein-interaction, microcontact printing, fluorescence microscopy, surface modification

## Abstract

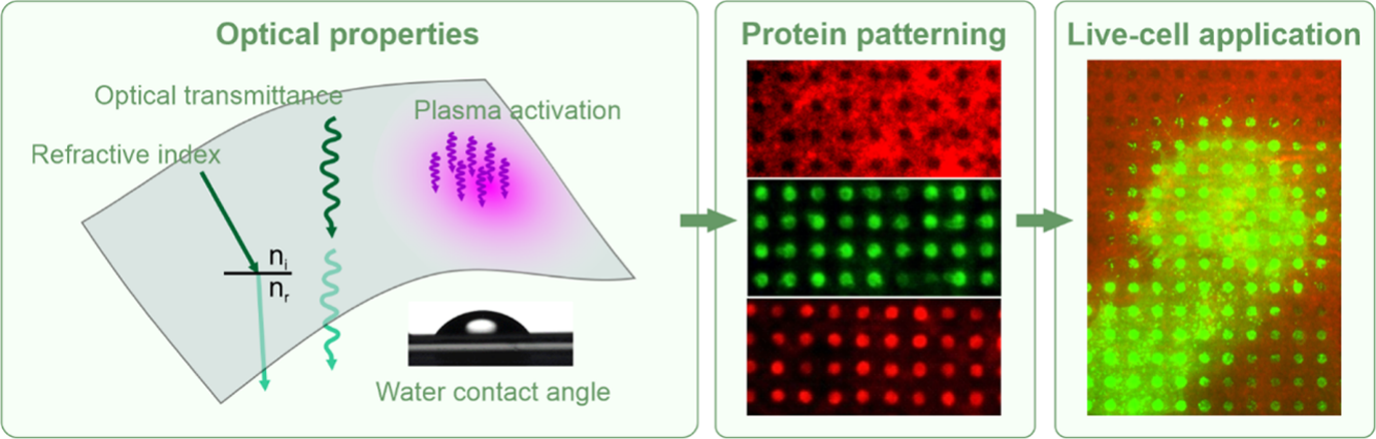

Polymeric materials play an emerging role in biosensing interfaces. Within this regard, polymers can serve as a superior surface for binding and printing of biomolecules. In this study, we characterized 11 different polymer foils [cyclic olefin polymer (COP), cyclic olefin copolymer (COC), polymethylmethacrylate (PMMA), DI-Acetate, Lumirror 4001, Melinex 506, Melinex ST 504, polyamide 6, polyethersulfone, polyether ether ketone, and polyimide] to test for the applicability for surface functionalization, biomolecule micropatterning, and fluorescence microscopy approaches. Pristine polymer foils were characterized via UV–vis spectroscopy. Functional groups were introduced by plasma activation and epoxysilane-coating. Polymer modification was evaluated by water contact angle measurement and X-ray photoelectron spectroscopy. Protein micropatterns were fabricated using microcontact printing. Functionalized substrates were characterized via fluorescence contrast measurements using epifluorescence and total internal reflection fluorescence microscopy. Results showed that all polymer substrates could be chemically modified with epoxide functional groups, as indicated by reduced water contact angles compared to untreated surfaces. However, transmission and refractive index measurements revealed differences in important optical parameters, which was further proved by fluorescence contrast measurements of printed biomolecules. COC, COP, and PMMA were identified as the most promising alternatives to commonly used glass coverslips, which also showed superior applicability in subcellular micropatterning experiments.

## Introduction

Due to their low cost and versatile properties, polymeric materials are nowadays one of the most utilized commercial products. Especially in the field of biosensor and lab-on-a-chip technologies, polymer substrates play an emerging role.^1^ Within this regard, polymers such as polydimethylsiloxane (PDMS), polycarbonate, polystyrene (PS), polymethylmethacrylate (PMMA), cyclic olefin copolymer (COC), and cyclic olefin polymer (COP) are used in microfluidic system fabrication.^2^ Because of their lack of functional chemical moieties for direct covalent attachment of biomolecules, those polymers cannot be used for biomolecule printing in an unmodified state. However, polymer substrates can be modified both chemically and topographically. Chemical methods to increase surface energy and to generate functional groups (such as carboxyl, amine, hydroxyl, or epoxy groups) include modifications such as plasma treatment, UV irradiation, and monolayer self-assembly.^3−5^ Topographical modification methods in the micron- and even submicron scale include photolithography,^6^ dip-pen lithography,^7^ laser-based “matrix assisted pulsed laser evaporation direct write” (MAPLE DW),^8^ microchannel cantilever spotting,^9^ and soft lithography via microcontact printing (μCP).^10^

Micropatterned biomolecules on different substrates have numerous biological applications in the fields of biomimetic sensors,^11^ microarrays,^12^ and lab-on-a-chip systems.^13^ On a cell-scale, microstructured biointerfaces have been extensively used to study the impact of extracellular cues on parameters such as cell polarization,^14^ morphology,^15^ endocytosis,^16^ migration,^17^ cytoskeleton dynamics,^18^ and differentiation.^19^ On a subcellular scale, micropatterned biomolecules on solid substrates have been applied to reorganize the distribution of membrane-bound and intracellular proteins to address different biological questions out of the field of receptor signaling kinetics,^20−22^ receptor complex formation,^23^ phagocytosis,^24^ endocytosis,^25^ plasma membrane organization,^26^ cell adhesion,^27^ receptor clustering,^28,29^ and even cytosolic protein complex formation.^30,31^

Microstructured biointerfaces are commonly fabricated on non-polymeric materials such as silicon or glass, which possess major drawbacks such as high fragility and increased specific costs. Hence, modified polymer foils with the ability to covalently bind biomolecules on the surface are of great interest. Different ways for covalent or non-covalent binding of biomolecules on modified polymer substrates have been reported.^32−36^ Within this regard, we applied biomolecule micropatterns on COP surfaces with adjustable contrast by means of a photolithographic approach for subcellular micropatterning experiments.^37^ However, the fabrication of biomolecule micropatterns by use of photolithography is time-consuming, requires clean room facilities, and expensive lab equipment. On the contrary, μCP is an appropriate alternative that is easy to implement, and no special lab devices are needed. Therefore, we have recently presented a method for the fabrication of large-area protein micropatterns on COP substrates by μCP to study the subcellular immunopatterning of cytosolic protein complexes.^30^ Besides the use of COP foils for fluorescence microscopy-based experiments, other polymer substrates might represent a valuable alternative for similar applications also allowing the mass fabrication of biosensor surfaces at low cost. However, most of the polymer substrates are not thoroughly examined concerning their optical properties and their suitability for printing of biomolecules and fluorescence microscopy applications.

Here, we describe and analyze the applicability of 11 different polymeric substrates for the fabrication of biomolecule micropatterns after chemical surface modification with functional groups and their suitability for epifluorescence and total internal reflection fluorescence (TIRF) microscopy. Furthermore, we demonstrate the ability of selected polymers for the quantitation of subcellular micropatterning experiments in living cells.

## Results and Discussion

### μCP as a Strategy to Produce Protein Patterned Polymer Substrates

We have recently introduced protein micropatterned COP foils as cost-saving and flexible alternatives to glass coverslips for subcellular micropatterning experiments by means of photo- and soft-lithographic approaches, respectively.^30,37^ COP foils were identified as superior substrates for patterning and covalent binding of biomolecules as well as for live cell experiments. However, there are several other polymers available, which might possess similar or even better properties than COP or glass coverslips. Therefore, we aimed in the investigation and comparison of 11 different polymers for their applicability in the fabrication of protein micropatterns, fluorescence microscopy, and subcellular micropatterning experiments. Polymer foils were preselected based on the main parameter which must be fulfilled for TIRF microscopy, a substrate thickness below 200 μm. Glass coverslips, still representing the standard substrate within this context, were used as a control surface throughout the study.

**Figure 1:**
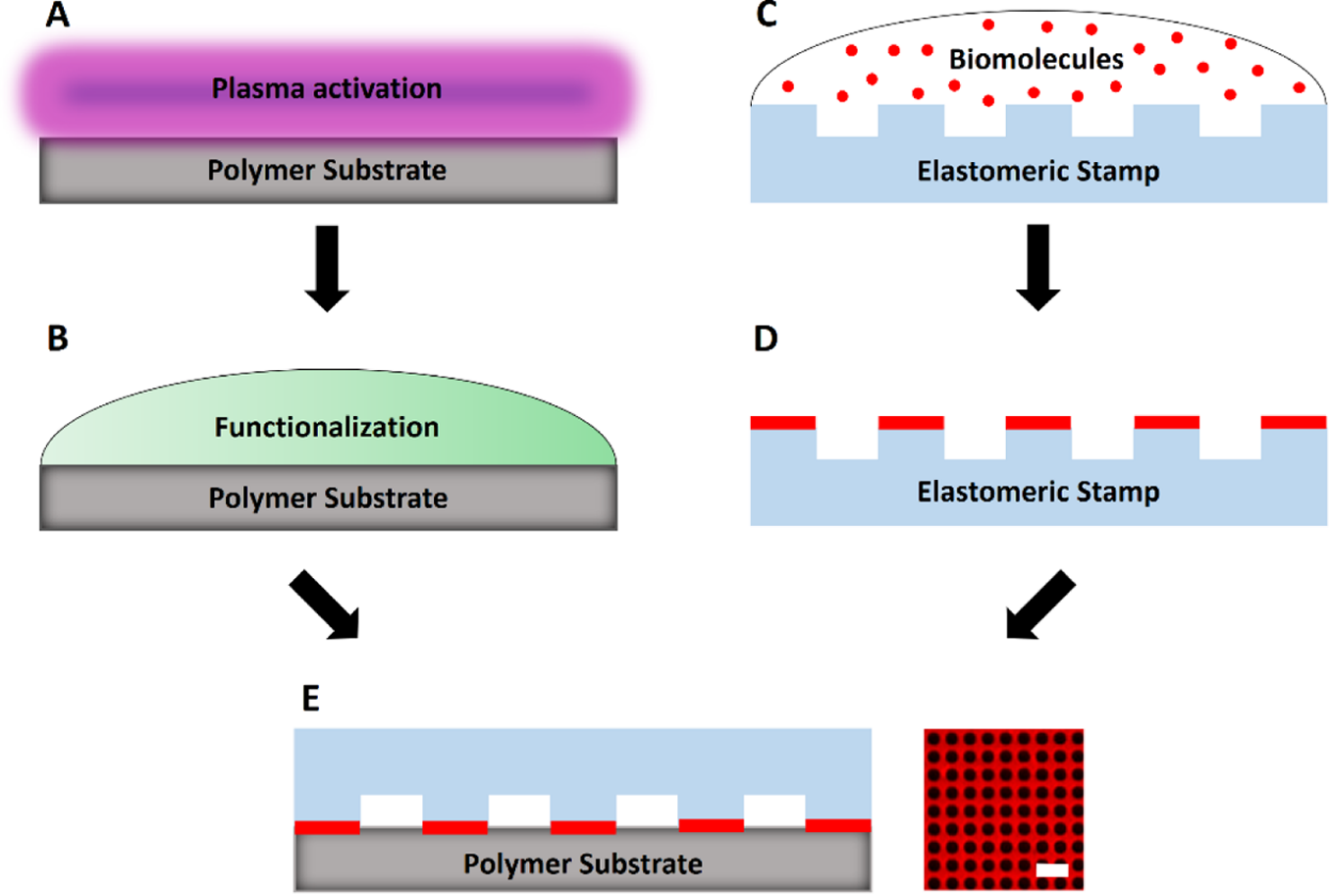
Schematic workflow of the preparation of BSA micropatterns by μCP on functionalized polymer substrates. In short, polymer foils are activated by air-plasma oxidation (A), followed by the introduction of epoxide functional groups (B). Next, a PDMS stamp with a feature size of 3 μm is incubated with the biomolecule solution (e.g., BSA or BSA-Cy5 for surface passivation) (C). After a washing step (D), the stamp is placed upside-down on the functionalized polymer substrate for biomolecule transfer (E). After stripping of the stamp, the patterned substrate is bonded with a 384-well plastic casting for stabilization of the flexible foil. Fluorescence micrograph shows representative TIRF microscopy image of a BSA-Cy5 patterned polymer substrate. Scale bar: 10 μm.

The general functionalization strategy used for all substrates under study is depicted in Figure 1. In an initial step, the polymer foils were air-plasma activated to introduce a substrate surface with a high density of oxygen-containing functional groups (Figure 1A). The activation state of polymer surfaces and subsequent protein immobilization was shown to be critically influenced by plasma treatment conditions and parameters such as plasma cycle time, compressed air flow rate, gas composition, substrate temperature, and power density.^38,39^ Additionally, we were able to create biomolecule micropatterns with adjustable contrast on COP surfaces by variation of process conditions during plasma activation.^37^ Based on that, we chose optimized conditions for polymer plasma activation and kept the settings constant in all experiments. Plasma activation was followed by a chemical treatment with 2% (v/v) glycidyloxypropyl trimethoxysilane (GPTS) solution to form a layer of epoxide functional groups on the surface (Figure 1B), as a prerequisite for subsequent covalent binding of biomolecules such as bovine serum albumin (BSA) or streptavidin. Protein micropatterns on epoxy-coated substrates were produced by μCP using microstructured PDMS stamps containing a grid pattern with a feature size of 3 μm. Briefly, the stamps were inked with a BSA-Cy5 (or just BSA) solution (Figure 1C), excess of liquid was removed by drying the stamp with nitrogen (Figure 1D), followed by the printing step on the freshly functionalized substrates, resulting in a BSA (-Cy5) patterned polymer surface with high spatial resolution (Figure 1E). Other approaches, such as UV-excimer laser photoablation^40^ or electron-beam-induced lithography,^41,42^ are possible alternatives for the fabrication of patterned biomolecule surfaces on a micro- and nanometer scale. However, soft lithography via μCP provides some unique features compared to such sophisticated photolithographic methodologies, which makes its application attractive. In general, the chemical structure and properties of the substrate and the stamp have a high impact on the transfer efficiency of the biomolecules during the printing process, whereas μCP of proteins is preferred from a low free energy stamp onto a high energy and hydrophilic surface.^43^ One of the biggest benefits of the μCP approach is that no special and expensive lab equipment is required. Furthermore, μCP is easy to implement and has a high level of robustness and reproducibility. Additionally, PDMS as a stamp material is favorable to other materials due to its hydrophobic surface. The low surface energy enables easy separation from the master during the fabrication process, reversible binding to the biomolecules during printing and simple peeling of the stamp from the substrate after the printing process. Furthermore, it is chemically inert, cheap, and the printing process itself is easy to perform.^43−45^

### Characterization of Polymer Surface Modification by Contact Angle Measurement

Static water contact angle measurements enable for the evaluation of the hydrophilic/hydrophobic properties of various surfaces.^46^Figure S2 shows the changes in water contact angle before the plasma treatment and after the introduction of epoxy groups. Pristine polymer foils exhibited water contact angles between 72 ± 2° (Melinex ST540) and 97 ± 3° (COC). In general, the hydrophilicity of the surfaces changed meaningful after the surface functionalization, as proved by a tremendous decrease of the water contact angles compared to conditions before plasma activation. The modified polymer substrates after GPTS treatment showed their hydrophilic nature yielding in water contact angles between 47 ± 2° [polyethersulfone (PES)] and 67 ± 2° (Melinex 506), with a mean value for all substrates being 56 ± 8°. This value is in line with what has been observed previously for GPTS SAMs on different surfaces.^47,48^ A commercially available epoxysilane-coated glass coverslip served as a control substrate, exhibiting a water contact angle of 50 ± 2°, which correlates with data described in other studies.^49,50^

### Optical Characterization of Different Polymer Foils

**Figure 2:**
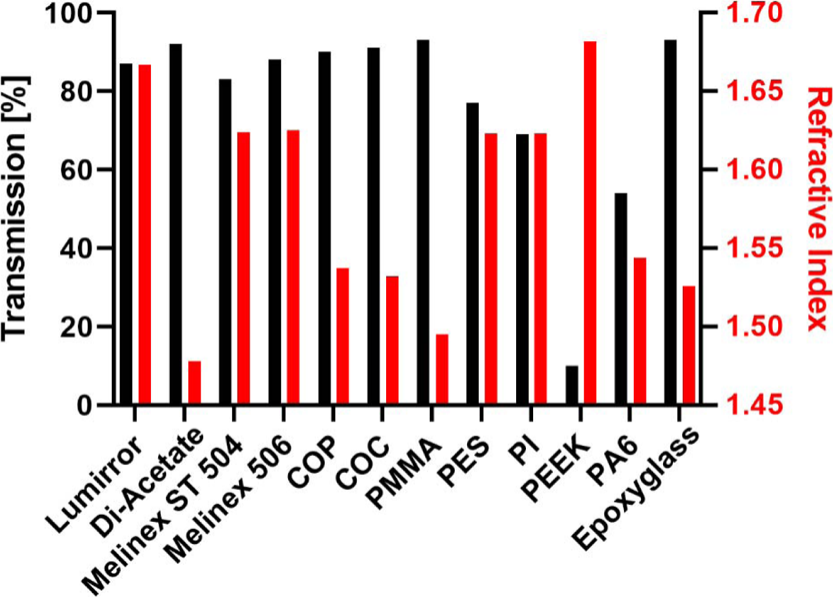
Transmission (black) and refractive index measurements (red) of different polymeric materials and epoxy-coated glass coverslips for substrate comparison.

We measured the optical transmittance spectra of the different polymer foils and a glass coverslip in the wavelength range from 200 to 800 nm (Figure S3) as an important parameter for fluorescence microscopy applications. Typical spectra of the excitation and emission of commonly used fluorophores are in the range between 400 nm and approximately 750 nm. The fluorescent dyes which we used for our subsequent protein patterning experiments, Cy5 and FITC, have their excitation maxima at 649 and 490 nm and their emission maxima at 666 and 525 nm, respectively. The transmittance of a typical optical glass used for fluorescence microscopy is ∼92% in the wavelength-range of interest.^51^ Therefore, a main prerequisite for the applicability of polymer substrates for fluorescence microscopy applications is a transmissivity comparable to commonly used microscopy glass coverslips. Peak transmission values for all substrates under study are shown in Figure 2 (black bars). COC, COP, PMMA, and DI-Acetate foils showed a transmittance in the measured wavelength range higher than 90%. Peak values of the transmittance spectra measured for Lumirror, Melinex 506, and Melinex ST504 were close to 90% (∼87, ∼88, and ∼82%, respectively). The lowest transmission values were obtained for polyether ether ketone (PEEK) (∼9%), polyamide 6 (PA6) (∼56%), polyimide (PI) (∼69%), and PES (∼78%) foils. For these polymers, the transmittance raised steadily with the increase in measurement wavelength. The low transmittance of these materials suggests that they might be not suitable for fluorescence microscopy applications.

In addition to the optical transmittance, we measured the refractive index of the different polymers as a second critical parameter for fluorescence microscopy (Figure 2, red bars), especially crucial for TIRF microscopy. Light is partially reflected and diffracted whenever it encounters the interface of two transparent media with different refractive indices.^52^ At the critical angle, that is given by Snell’s law, light is completely reflected and total internal reflection occurs.^53^ This phenomenon can be observed when light travels from a medium with higher refractive index to a medium with a lower one. Hence, the refractive index of the solid substrate plays an important role in TIRF microscopy.^54^ Control glass coverslips exhibited a refractive index of 1.53, which is in line with the reported optical properties of the supplier (Schott). COC, COP, PMMA, PA6, and DI-acetate foils showed similar refractive index values when compared to the glass coverslip (1.53, 1.54, 1.5, 1.55, and 1.48, respectively). For all other polymers, the refractive index was higher and ranged between 1.62 and 1.68. All tested substrate foils showed a higher refractive index than the standard aqueous cell culture medium and buffers (*n* ∼ 1.33),^55^ which were used for cell cultivation and for the subsequent fluorescence microscopy evaluations. Thus, the prerequisite for TIRF microscopy is fulfilled for all polymer foils.^56^ However, not only the substrate refractive index but also the refractive indices from the media and the sample, the wavelength of the used excitation light, and the numerical aperture of the objective influence the depth of penetration of the evanescent wave and therefore the quality of TIRF images.^53,56^

### Characterization of Protein Patterned Substrates via Epifluorescence Microscopy

**Figure 3:**
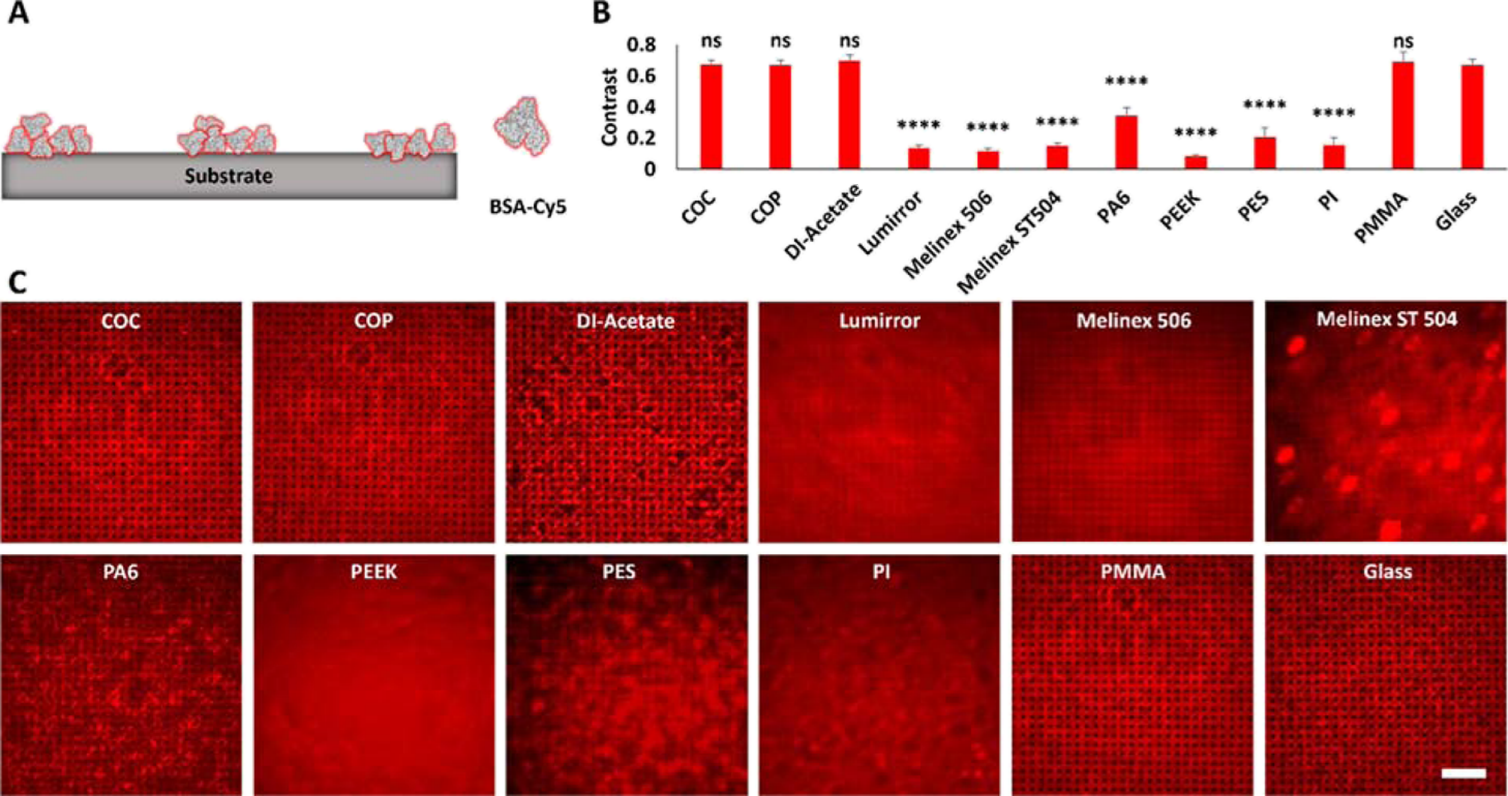
Characterization of the fluorescence pattern contrast of printed BSA-Cy5 on various substrates. (A) Schematic overview of Cy5-labeled BSA printed to the substrate surface. (B) Quantitation of the BSA-Cy5 fluorescence contrast. (C) Representative epifluorescence microscopy images of indicated BSA-Cy5 printed polymer foils. Scale bar: 30 μm. Contrast values are presented as mean ± standard deviation. *n* = 7; ****p < 0.0001 for comparison of fluorescence contrast with glass substrate; ns, no significant difference.

To elaborate on the applicability of the polymers under study for fluorescence microscopy, plasma activated, and GPTS-treated foils were further functionalized by protein patterning (BSA-Cy5) via PDMS-based μCP (as schematically depicted in Figure 1). The transfer of the micron-scale BSA-Cy5 grid onto the polymer substrates was first characterized using epifluorescence microscopy with a 20× air objective (Figure 3). The fluorescence contrast ⟨*c*⟩ between BSA-Cy5 patterned and non-decorated (still active) areas was used to evaluate and compare the quality of protein transfer as well as the general applicability for fluorescence microscopy (Figure 3A,B). The calculated fluorescence BSA-Cy5 contrast for the control glass coverslip was ⟨*c*⟩ = 0.67 ± 0.04, which was even excelled by the polymer substrate DI-acetate (⟨*c*⟩ = 0.70 ± 0.04), although some patchy grid structures were obtained in restricted areas, which might be explained by beginning degradation processes of the cellulose acetate-based material. Three additional polymer substrates exhibited similar contrast values similar to the glass coverslip (⟨*c*^COP^⟩ = 0.67 ± 0.03, ⟨*c*^COC^⟩ = 0.67 ± 0.03 and ⟨*c*^PMMA^⟩ = 0.69 ± 0.06). We could recently show that μCP of BSA results in the formation of a BSA monolayer on glass substrates with an average height of ∼3–4 nm,^57^ which is likely to be the same for polymer substrates showing comparable BSA-Cy5 fluorescence contrast values. Significantly lower contrast values were obtained for PA6 (⟨*c*⟩ = 0.35 ± 0.05) and PES (⟨*c*⟩ = 0.21 ± 0.06), whereas a further reduction was observed for all other polymers (⟨*c*^Lumirror^⟩ = 0.14 ± 0.02, ⟨*c*^Melinex 506^⟩ = 0.12 ± 0.02, ⟨*c*^Melinex ST504^⟩ = 0.15 ± 0.02, ⟨*c*^PEEK^⟩ = 0.08 ± 0.01, and ⟨*c*^PI^⟩ = 0.16 ± 0.05). Representative epifluorescence images of all substrates are shown in Figure 3C. Except for PA6, the reduced ⟨*c*⟩ might be explained not only by a lower BSA-Cy5 grid quality and decreased protein transfer but also with encountered light scattering effects, which indicates low suitability for fluorescence microscopy or for further application in TIRF microscopy. On the one hand, polymers with low transmission values such as PEEK might not be suitable for fluorescence microscopy approaches at all, but on the other hand, also some polymers with good transmission properties such as Lumirror and Melinex foils showed low fluorescence contrast values. Within this regard, other material properties such as impurities, inclusions, pores, or general effects in grain boundaries may cause light scattering effects leading to low fluorescence contrast in our quantitative analysis regime. Local polymer network defects, density fluctuations, and spatial inhomogeneities may also intensify the effect of light scattering and contribute to shortcomings in the optical quality of certain polymer substrates.^58,59^ Furthermore, we could show that the fluorescence contrast ⟨*c*⟩ is inversely correlated with the overall global background fluorescence of functionalized foils, which might be an additional explanation for the obtained differences (Figure S4). Polymers such as COC, parylene C, PS, and PDMS have already been reported for the fabrication of protein micropatterns by a modified μCP approach and were characterized via fluorescence microscopy. In this study, the protein surface concentration was adjusted by varying both the concentration in the incubation solution and the incubation time, respectively.^60^ However, for reasons of comparability, we kept these parameters constant during our experiments (based on parameters optimized in previous studies^20,61^). Furthermore, three-dimensional plasma micro-nanotextured COP surfaces for biomolecule immobilization were recently reported.^62^ Apart from generating biomolecule micropatterns on modified polymer substrates, other advantages compared to glass or silicon surfaces should be highlighted: physical and chemical surface modification techniques can be used for bonding polymers at high bond strength without the use of strong solvents or high temperature. These and other bonding methods can be easily implemented in microfluidic chip fabrication.^63,64^

### Characterization of Selected Protein Patterned Substrates via TIRF Microscopy

**Figure 4:**
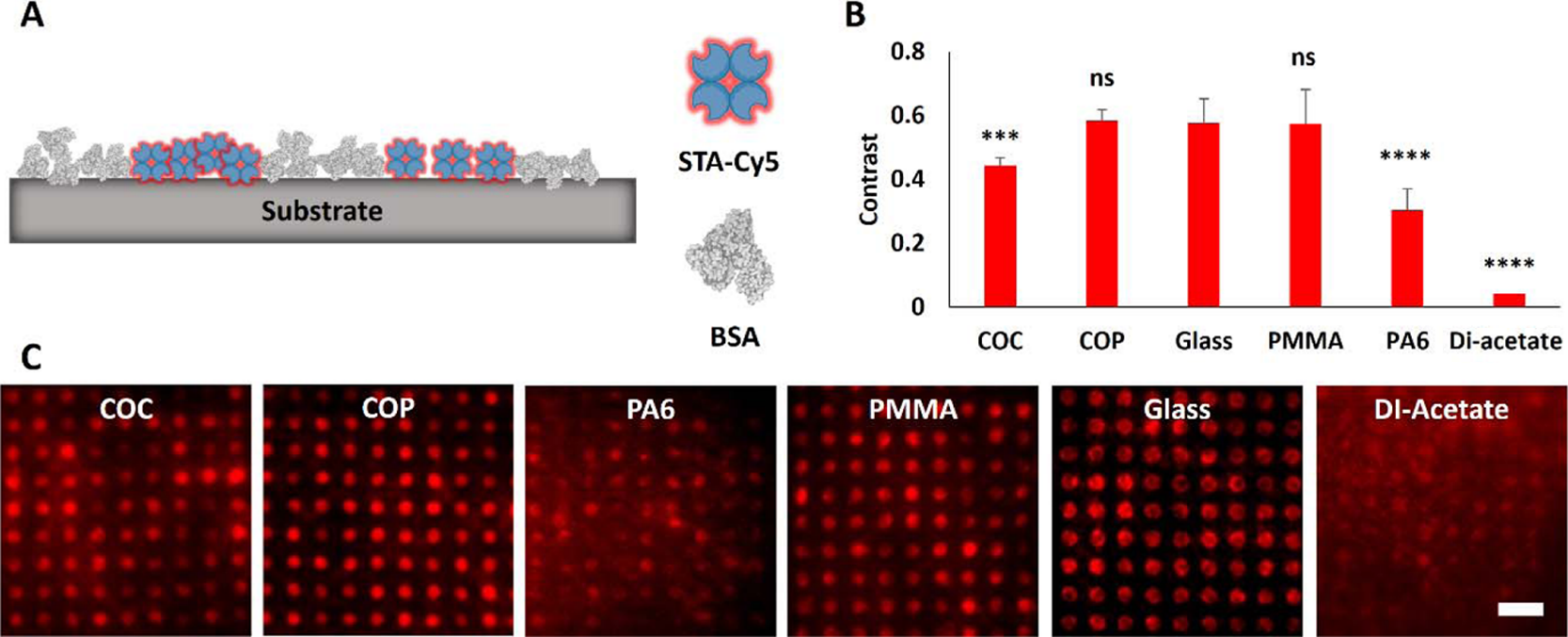
Characterization of the fluorescence pattern contrast of incubated streptavidin-Cy5 (STA-Cy5) on various BSA-patterned substrates. (A) Schematic overview of STA-Cy5 bound to the activated areas of the substrate surface. (B) Quantitation of the STA-Cy5 fluorescence contrast. (C) Representative TIRF microscopy images of indicated STA-Cy patterned polymer foils. Scale bar: 10 μm. Contrast values are presented as mean ± standard deviation. *n* = 7; ****p < 0.0001, and ***p < 0.001 for comparison of fluorescence contrast with glass substrate; ns, no significant difference.

Next, polymer substrates were further characterized using TIRF microscopy. Polymers with inappropriate features (low fluorescence transmittance, high refractive index compared to glass, and general light scattering effects) were excluded from further protein patterning experiments. In general, the refractive index of a surface substrate used for TIRF microscopy should be as close as possible to the one of the used immersion oil in order to avoid reflection and deflection effects.^65^ Therefore, we proceeded with following substrates: COC, COP, DI-Acetate, PA6, and PMMA (Figure 4). To further elaborate on the BSA passivation efficiency of our μCP procedure and on the implementation of the covalent streptavidin–biotin binding system (as a prerequisite for further subcellular live cell studies), BSA-patterned substrates were functionalized with Cy5-labeled streptavidin (Figure 4A). Again, the fluorescence contrast ⟨*c*⟩ served as a quality parameter for protein transfer and binding (Figure 4B). Image analysis confirmed the general eligibility of all selected polymer foils for TIRF microscopy (Figure 4C). COC, COP, and PMMA patterned foils delivered comparable ⟨*c*⟩ values as glass coverslips (⟨*c*^COP^⟩ = 0.59 ± 0.03, ⟨*c*^COC^⟩ = 0.44 ± 0.03, ⟨*c*^PMMA^⟩ = 0.57 ± 0.11 and ⟨*c*^glass^⟩ = 0.58 ± 0.08), whereas PA6 resulted in a significantly lower ⟨*c*⟩ (0.3 ± 0.07), which is in line with the observed reduced BSA-Cy5 transfer (Figure 3). Interestingly, DI-acetate showed unfavorable properties concerning its stability when incubated with biomolecules in liquidity. A time-dependent degradation process was observed with values decreasing to ⟨*c*⟩ 0.04 ± 0.01 after 30 min of incubation (Figure S5). Therefore, DI-acetate was found not to be a suitable substrate for subsequent TIRF microscopy experiments and was not considered for further characterization. The degradation process of cellulose acetate-based materials is well known but the presence of additives in the polymer composition influences the degradation mechanism and rate. Besides biological degradation, photo-degradation processes might play an additional role.^66^

Additionally, X-ray photoelectron spectroscopy (XPS) measurements were carried out on the most promising polymer materials COC, COP, PA6, and PMMA before and after functionalization in order to monitor the chemical changes of the individual surfaces and to directly compare the amount of the deposited GPTS. In Figure S6, XPS survey spectra before and after GTPS-functionalization are depicted for the COP foil material as example. Whereas oxygen can be found on the pristine sample only in small traces as surface contaminant, the content of oxygen as well as silicon significantly increased after the treatment. A summary of the elemental surface composition before and after functionalization can be found in Table S1, clearly showing concentrations of silicon up to 1.7 at. % arising from GPTS after the treatment. A detailed view on the original and added chemical functionalities is given in Figure S7 for these four polymer materials in the high-resolution (HR) XPS spectra of the C 1s, O 1s, as well as of the Si 2p photoelectron peaks after successful GPTS-functionalization.

**Figure 5:**
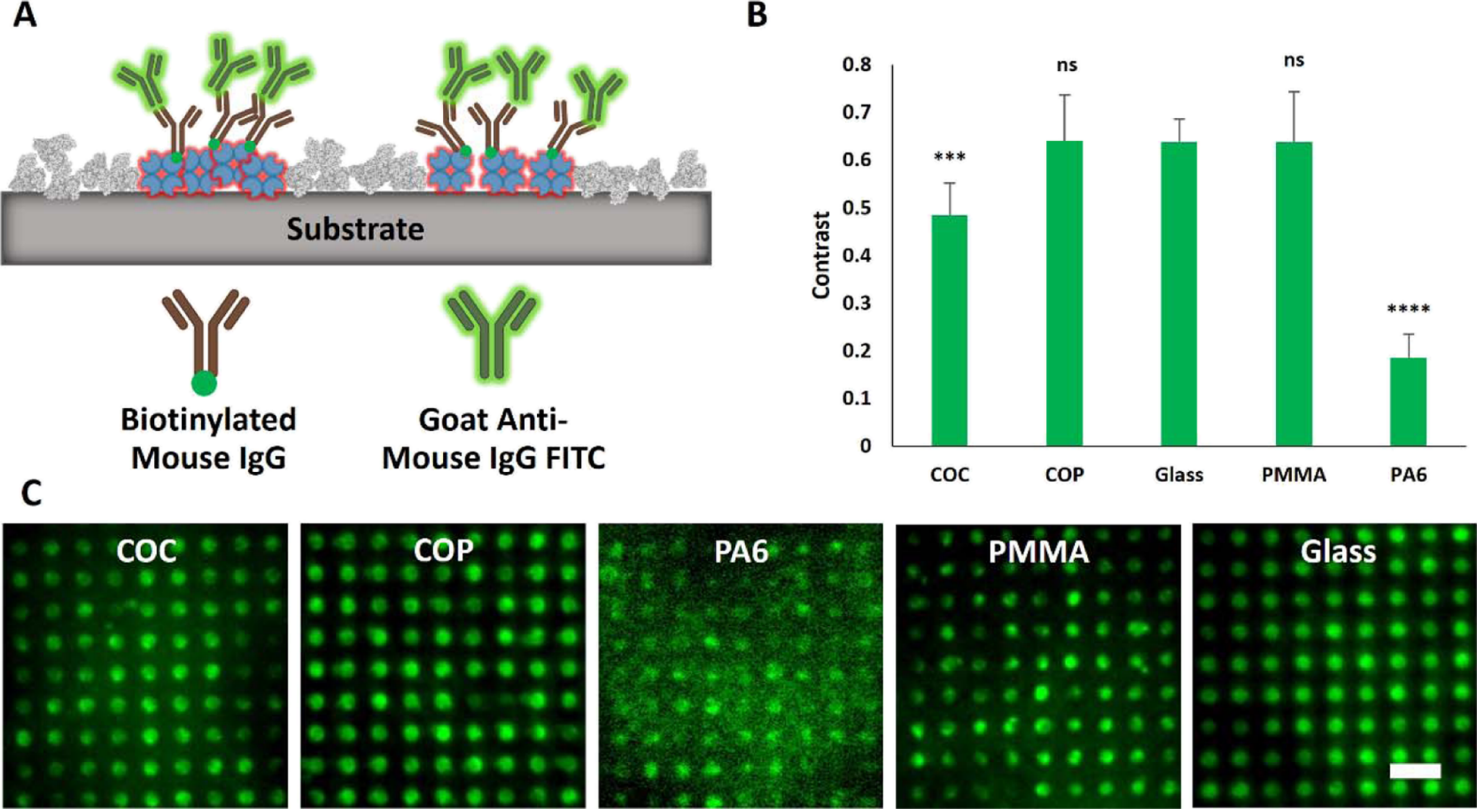
Characterization of the fluorescence pattern contrast of incubated FITC-Ab on various BSA-patterned substrates further functionalized with STA. (A) Schematic overview of micropatterned biotinylated mouse IgG detected with FITC-labeled anti-mouse IgG antibody. (B) Quantitation of FITC-Ab fluorescence contrast. (C) Representative TIRF microscopy images of indicated FITC-Ab patterned polymer foils. Scale bar: 10 μm. Contrast values are presented as mean ± standard deviation. *n* = 7; ****p < 0.0001, and ***p < 0.001 for comparison of fluorescence contrast with glass substrate; ns, no significant difference; ns: no significant difference.

Subcellular micropatterning experiments require the spatial reorganization of membrane proteins into an ordered array according to the microstructured substrate. This can be achieved by different means, most easily by the use of respective antibodies against a membrane protein of interest.^29,67,68^ Remaining polymer substrates (decorated with micron-scale BSA grid and streptavidin patterns, as shown in Figure 4) were therefore further functionalized using biotinylated primary as well as fluorescently labeled secondary antibodies (Figure 5). A schematic overview of the resulting micropatterned antibody surface is shown in Figure 5A. The above-detected differences in streptavidin surface binding correlated well with the subsequent binding capacity of antibodies, with PA6 delivering a significantly lower ⟨*c*⟩ (0.19 ± 0.05) compared to the remaining substrates (⟨*c*^COP^⟩ = 0.64 ± 0.10, ⟨*c*^COC^⟩ = 0.49 ± 0.07, ⟨*c*^PMMA^⟩ = 0.64 ± 0.10, and ⟨*c*^glass^⟩ = 0.64 ± 0.05) (Figure 5B,C). Based on this result, PA6 was not used for final proof-of-concept cell patterning experiments. Furthermore, as already described and characterized in our previous studies,^37,61^ glass coverslips, and COP foils were also excluded.

### Subcellular Micropatterning of Artificial Receptors in the Live Cell Membrane

**Figure 6:**
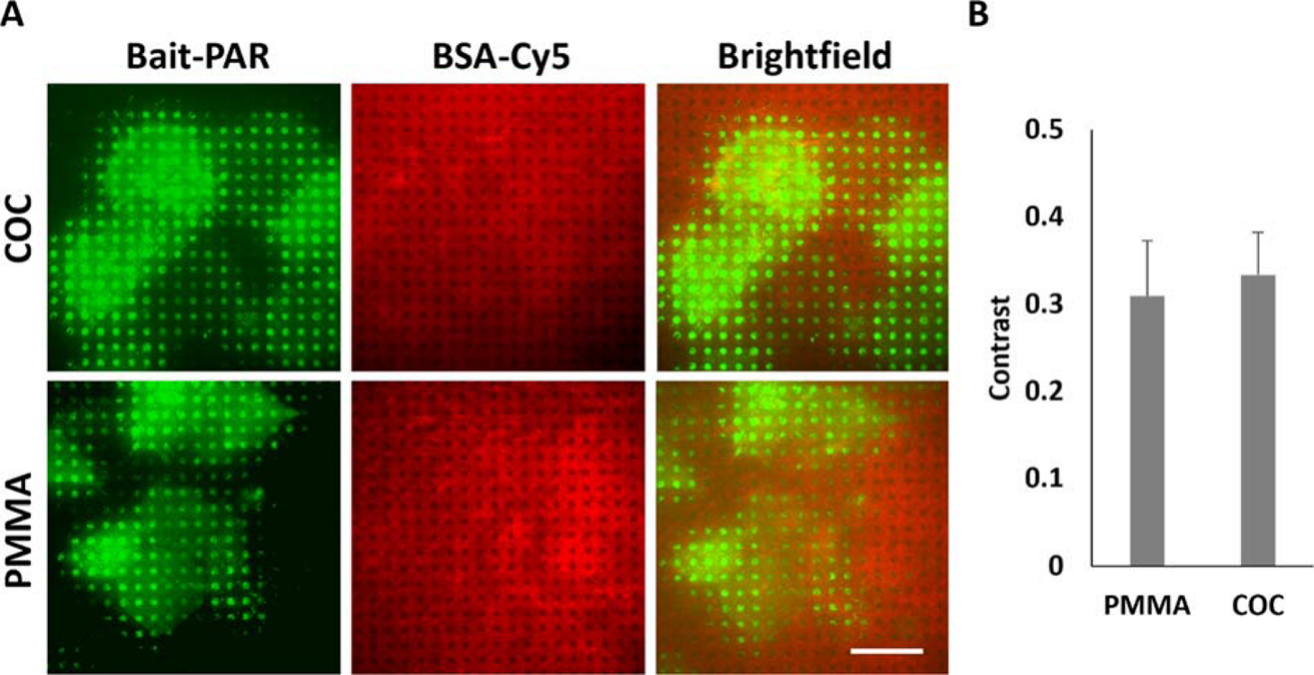
Applicability of selected polymer substrates for subcellular micropatterning experiments. (A) Hela cells transiently expressing the recently published GFP-fused bait-PAR-Grb2 (30) were grown on anti-HA antibody patterned polymer COC and PMMA substrates. Representative TIRF microscopy images bait-PAR-Grb2 expressing cells (green, left), BSA-Cy5 printed grid (red, middle), and the respective brightfield image of the adhered cells (gray, right). Scale bar: 30 μm. (B) Quantitation of fluorescence contrast. Data represented mean ± standard deviation of 25 analyzed cells measured in two different experiments.

We have recently established an approach for subcellular dynamic immunopatterning of cytosolic proteins by use of an artificial transmembrane bait construct (bait-PAR).^30^ In order to demonstrate the applicability of COC and PMMA substrates for live cell experiments, cells expressing a GFP-fused bait-PAR [containing a human influenza hemagglutinin (HA) epitope tag] were grown on anti-HA antibody patterned surfaces (Figure 6). Upon a specific antibody–antigen interaction, bait-PARs were rearranged in the plasma membrane according to the micrometer-scale antibody pattern on the respective substrate (Figure 6A), proving on the one hand the biocompatibility and on the other hand the applicability for cell micropatterning experiments of these polymer materials. The fluorescence contrast ⟨*c*⟩ served again as a parameter for the suitability of these polymer materials for TIRF microscopy in live cell experiments (Figure 6B). There was no significant difference in fluorescence contrast of COC and PMMA (⟨*c*^COC^⟩ = 0.33 ± 0.05, ⟨*c*^PMMA^⟩ = 0.31 ± 0.06). These two substrates gave equivalent results compared to COP and glass substrates which are used in other studies with similar surface technology and comparable biological systems.^30^ Our data suggest that COC and PMMA substrates are suitable alternatives to glass and COP surfaces for TIRF microscopy.

## Conclusions

In this study, we describe an extensive optical characterization of different polymer materials for biomolecule immobilization, quantitative fluorescence microscopy, and subsequent live cell micropatterning experiments. Eleven different substrates and commercially available glass coverslips as a reference were tested. We first analyzed optical properties and surface characteristics of selected materials via spectrophotometry, refractive index, and contact angle measurements. Furthermore, we demonstrated the possibility for the introduction of functional groups onto all selected polymer substrates as a prerequisite for biomolecule patterning via μCP. COC, COP, and PMMA substrates turned out to be feasible and flexible alternatives to glass substrates with tunable chemical and comparable optical properties. Furthermore, the benefits of polymer foils over glass cover slips give a competitive advantage in manufacturing processes when scaling-up the fabrication of biointerfaces.

## Materials and Methods

### Materials

BSA, streptavidin, 3-GPTS (98%), biotinylated mouse IgG antibody, and PDMS (SYLGARD 184) were purchased from Sigma Aldrich (Schnelldorf, Germany). FITC-labeled goat anti-mouse IgG antibody and anti-HA tag antibody were obtained from antibodies-online GmbH (Aachen, Germany). BSA-Cy5 was purchased from Protein Mods (Madison, WI, USA). Photolithographically patterned wafer were obtained from Delta Mask B.V. (Enschede, Netherlands). Epoxy-functionalized NEXTERION glass coverslips (24 mm × 50 mm, 175 ± 20 μm thickness) were from Schott GmbH (Jena, Germany). COP (Zeonor) foil with a thickness of 100 μm was obtained from microfluidic ChipShop GmbH (Jena, Germany). COC and PMMA foils were a kind gift from DENZ Bio-Medical GmbH (Oberwart, Austria). Melinex 506 (polyester film), Melinex ST 504 (polyester film), DI-acetate (cellulose acetate), and Lumirror 4001 (polyester film) foils were provided from Pütz GmbH (Taunusstein, Germany). PA6, PEEK, PI, and PES substrates were obtained from RCT Reichelt Chemietechnik GmbH (Heidelberg, Germany).

### Optical Analysis

Optical transmission spectra of pristine polymer foils were measured with a step size of 5 nm in the wavelength range from 200 to 800 nm using a Cary 60 UV–vis spectrophotometer (Agilent Technologies, Santa Clara, CA, USA). Refractive Index was measured by means of an Abbe refractometer, where the film samples were inserted between the two glass prisms with diiodomethane as contact liquid for all films except for the Melinex-samples, where carbon disulfide was used, as diiodomethane did alter the surface of the samples in the tests.

### Substrate Functionalization

Polymer substrates were washed with ethanol and dH_2_O before drying with nitrogen and hydrophilization by plasma oxidation in a commercially available hot wall plasma-assisted chemical vapor deposition reactor (Rübig GmbH, Wels, Austria). The oxidation process was performed with O_2_ regulated by a mass flow controller for 10 min. The pressure was obtained by means of a screw pump in combination with a roots blower and controlled by a throttle valve at a pressure of 80 Pa. The deposition temperature was 298 K at a power density of 100 W/m^2^. Hydrophilized polymer foils were subsequently incubated overnight in 2% (v/v; in dH_2_O) GPTS solution to form a layer of epoxide functional groups on the surface. As a last step, the polymer substrates were washed with ethanol and dried with nitrogen. Substrates were stored at 4 °C or immediately used for μCP.

### Preparation of Biomolecule Micropatterns by μCP

PDMS stamps were fabricated by mixing PDMS prepolymer in a ratio of 10:1 (w/w, precursor/curing agent) and degassing of the mixture in order to remove air bubbles in a desiccator for 30 min. The PDMS mixture was poured on a silanized wafer containing an array of round-shaped pillars with a feature size and depth of 3 μm. The mixture on the wafer was degassed again, cured for 2 h at 80 °C, and finally peeled off from the wafer. The micron-scale surface of the PDMS stamp is shown in Figure S1A.

For μCP, the PDMS stamp was washed with ethanol and dH_2_O before drying with nitrogen. The stamp was incubated with a BSA or BSA-Cy5 solution (1 mg/mL) for 30 min at room temperature in the dark. The stamp was washed again with phosphate-buffered saline (PBS) and dH_2_O and dried under a stream of nitrogen. The stamp was placed upside down by its own weight on the functionalized polymer substrate and incubated at 4 °C overnight. This step was followed by peeling the stamp off from the substrate and the bonding of the micropatterned foil (Figure S1B) to a 384-well plastic casting using an adhesive tape (3M). Reaction chambers were subsequently incubated with 20 μL of streptavidin or streptavidin–Cy5 solution (50 μg/mL) for 30 min at room temperature. After washing the chamber three times with PBS, 20 μL of biotinylated antibody solution (10 μg/mL) was incubated for another 30 min, followed by another washing step with PBS. As a last step, 20 μL of fluorescently labeled secondary antibody solution was incubated for 30 min. Fluorescently labeled biomolecule micropatterns were imaged by fluorescence microscopy.

### Contact Angle Measurement

Water contact angle was measured at room temperature with a Krüss DSA30 contact angle instrument (Hamburg, Germany) following the sessile drop method with 5 μL of water. Each reported contact angle represents an average value of at least five separate measurements of a certain polymer. The measurements were performed on pristine and on functionalized polymer substrates.

### X-ray Photoelectron Spectroscopy

XPS data were recorded with a Thetaprobe XPS device (Thermo Scientific, UK), which is operated and controlled by the Avantage software package from the system supplier. The device is equipped with a monochromated Al K_α_ X-ray source (*h*ν = 1486.6 eV) and a dual flood gun for neutralizing the surface charge. The X-ray spot on the sample surface had a diameter of 400 μm. Survey (overview) spectra were recorded using a pass energy of 200 eV and an energy step width of 1 eV, while detailed HR spectra were obtained with 20 eV pass energy and 0.05 eV step width. The obtained spectra were charge corrected with respect to the C 1s peak of C–C/H similar to carbon at a binding energy of 285.0 eV.

### Scanning Electron Microscopy

Scanning electron microscopy (SEM) images of PDMS stamps and of micropatterned biomolecule surfaces on polymer substrates were obtained by coating the sample with gold directly before imaging with a scanning electron microscope TESCAN MIRA3 (Brno, Czech Republic). The polymer samples were mounted on aluminum cylinder stubs of 25 mm diameter and 3 mm height using self-adhesive carbon conductive tabs. The acceleration potential used during the investigation was 10 keV.

### Fluorescence Microscopy

Fluorescence microscopy was performed using an epi-fluorescence microscope Olympus IX81 equipped with suitable filter sets. Diode lasers were used for fluorescence excitation at appropriate wavelengths (Toptica Photonics, Munich, Germany). Epi-fluorescence signals were measured using a 20× objective (Olympus UPlanFL N 20×). For TIRF microscopy, samples were illuminated in total internal reflection configuration (CellTIRF, Olympus) using a 60× oil immersion objective (NA = 1.49, APON 60XO TIRF, Olympus, Munich, Germany). For the detection of fluorescence, a charge-coupled device camera (Orca-R2, Hamamatsu, Japan) was used. Samples were mounted on an *x*–*y*-stage (CMR-STG-MHIX2-motorized table; Märzhäuser, Wetzlar, Germany) and scanning of larger areas was supported by a laser-guided automated focus-hold system (ZDC-2; Olympus).

### Cell Culture and Transfection

HeLa cells were obtained from ATCC and cultured in a RPMI medium supplemented with 10% fetal bovine serum and 1% penicillin/streptomycin (all PAN-Biotech GmbH, Aidenbach, Germany) and grown at 37 °C in a humidified incubator with 5% CO_2_. Cells were transiently transfected with bait-PAR-Grb2 plasmid (30) using jetOPTIMUS DNA transfection reagent (Polyplus transfection, Illkirch, France) according to the manufacturer’s protocol.

### Subcellular Micropatterning Experiments

Selected reaction chambers from the 384-well plate were incubated with 20 μL of streptavidin for 30 min, washed three time with PBS, followed by incubation with biotinylated anti-HA antibody for 30 min at room temperature. The chambers were washed again three times with PBS and cells transiently expressing GFP-fused bait-PAR-Grb2 were seeded at the surface for at least 3–4 h before live cell microscopy analysis.

### Image Analysis and Statistical Analysis

The results are expressed as the mean ± standard deviation unless stated otherwise. Image processing and analysis was performed using ImageJ (NIH, Bethesda, MD, USA) and Microsoft Office Excel 365 (Redmond, WA, USA). The contrast of fluorescent biomolecule patterns was analyzed and described previously^61^ using the following formula: ⟨*c*⟩ = (F^+^ – F^–^)/(F^+^ – F_BG_). F^+^ and F^–^ represent the fluorescence intensity in the inner and surrounding pixels of the pattern, respectively. F_BG_ represents the global fluorescence background, which refers to a micropatterned polymer foil not incubated with fluorescent molecules. Fluorescence contrast analysis of subcellular micropatterning experiments was performed using the Spotty framework as described previously.^20,30^ An unpaired *t*-test was used for significance testing.

## Supporting Information Available

SEM images of a PDMS stamp and BSA micropatterned COP foil; water contact angle measurements of polymer substrates under study; transmittance spectra of pristine polymer substrates; quantitation of the global fluorescence background of GPTS-functionalized polymers; time-dependent degradation process of BSA-patterned DI-acetate substrates; XPS survey spectra of COP foils; HR XPS spectra of selected polymers; and elemental surface composition of selected polymer foils (PDF)
