# Supplementary material for: Microcontact Printing of Biomolecules on Various Polymeric Substrates: Limitations and Applicability for Fluorescence Microscopy and Subcellular Micropatterning Assays
Source: ACS Appl Polym Mater. 2022 Sep 6;4(10):6887–96. doi: 10.1021/acsapm.2c00834 (PMC9578008; doi:10.1021/acsapm.2c00834)
Supplement: Supplementary file 1 — ap2c00834_si_001.pdf [file ap2c00834_si_001.pdf]

Supporting Information

Microcontact printing of biomolecules on various polymeric substrates: limitations and applicability for fluorescence microscopy and subcellular micropatterning assays

Roland Hager[1*], Christian Forsich[1], Jiri Duchoslav[2], Christoph Burgstaller[3], David Stifter[2], Julian Weghuber[1, 4] and Peter Lanzerstorfer[1*]

[1] University of Applied Sciences Upper Austria, School of Engineering, 4600 Wels, Austria
[2] Center for Surface and Nanoanalytics (ZONA), Johannes Kepler University Linz, 4040 Linz, Austria
[3] Transfercenter fuer Kunststofftechnik GmbH, 4600 Wels, Austria
[4] FFoQSI - Austrian Competence Center for Feed and Food Quality, 3430 Tulln, Austria

* Correspondence to: peter.lanzerstorfer@fh-wels.at

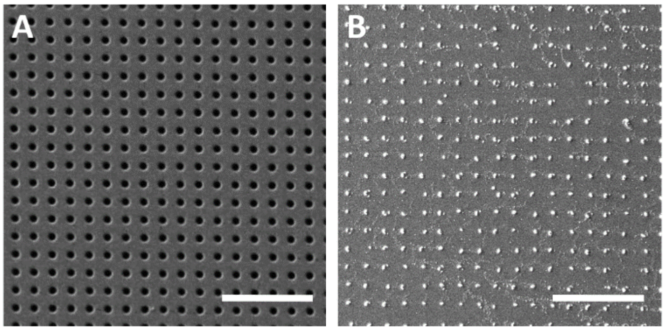

Figure S1. Scanning electron micrograph of PDMS stamp (A) and micropatterned BSA molecules on functionalized COP foil (B). Scale bar: 30 μm.

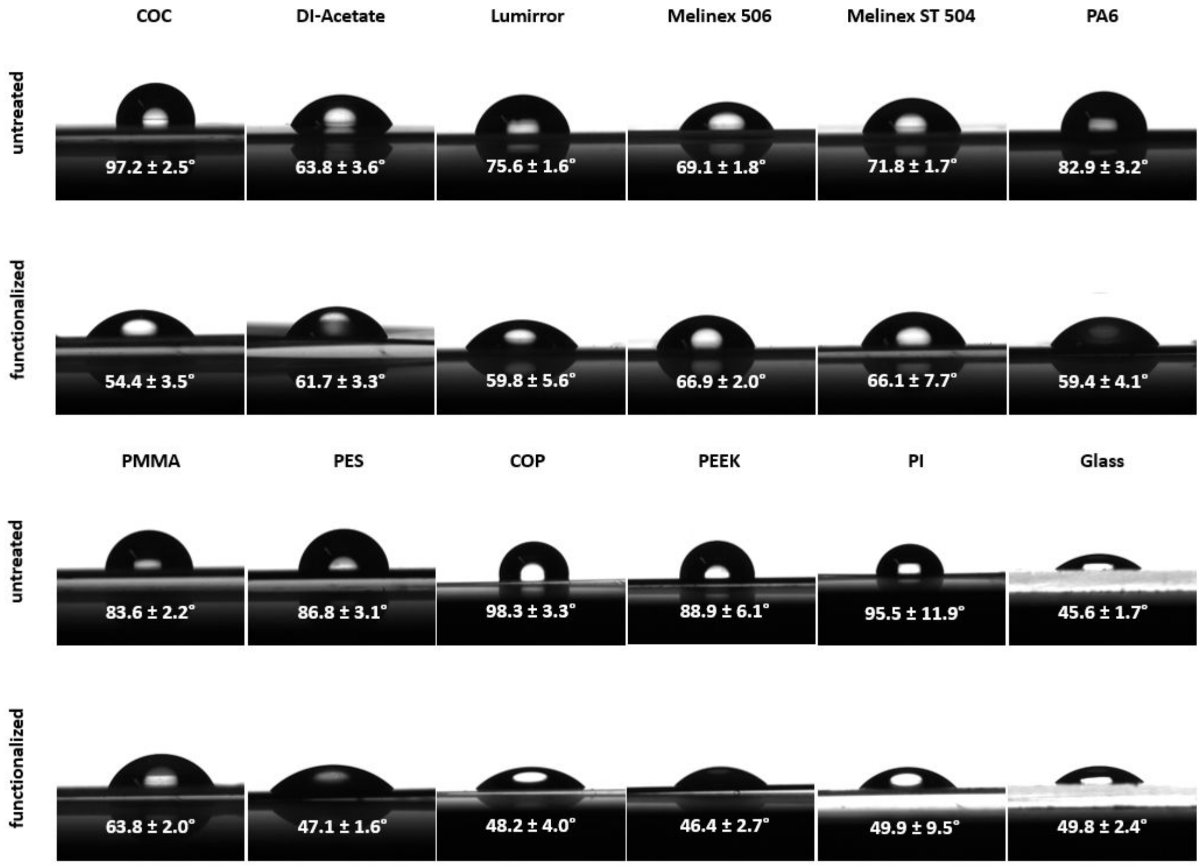

Figure S2. Measurement of static water contact angle of different polymer substrates before and after plasma treatment and functionalization with epoxy groups. Water contact angles after plasma treatment of polymer foils under study ranged between 0-10° (not shown).

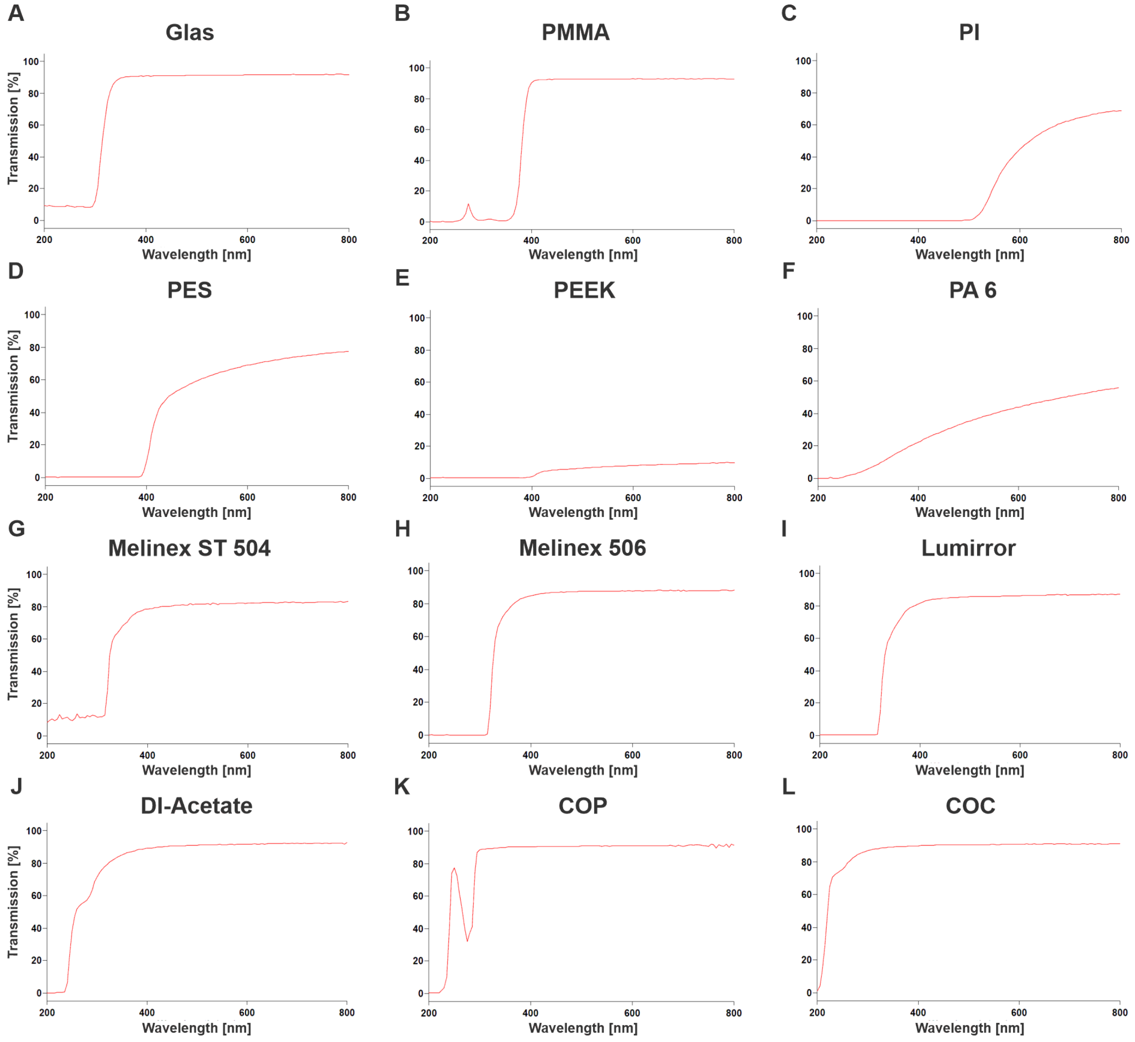

Figure S3. Transmittance spectra of pristine polymer substrates of indicated materials (A-L).

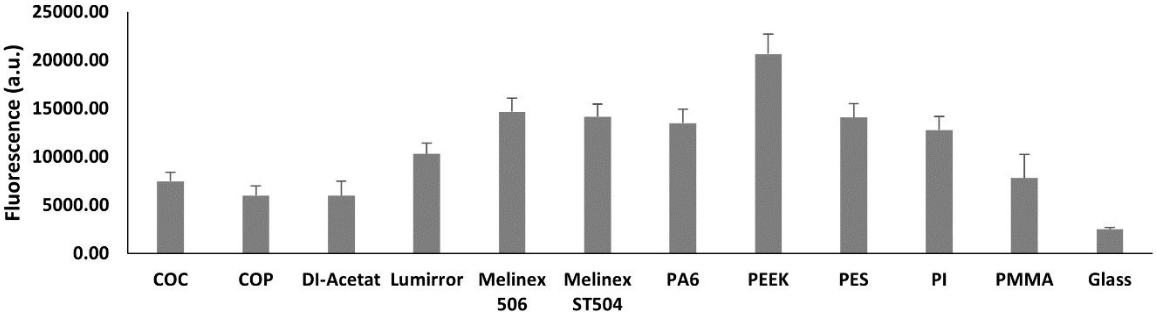

Figure S4. Quantitation of global fluorescence background of GPTS-functionalized polymers.

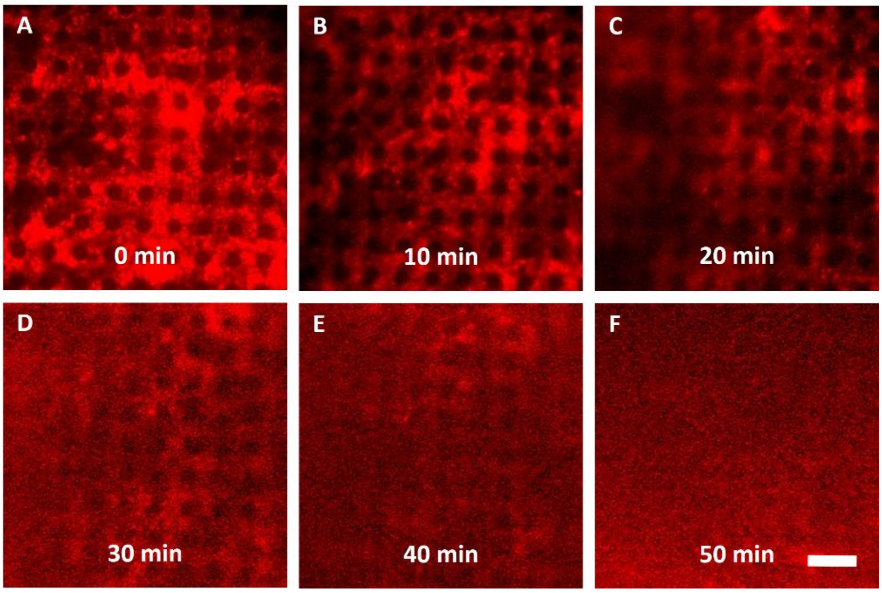

Figure S5. Time-dependent degradation process of BSA-patterned DI-acetate substrate after wetting with PBS. TIRF microscopy images at different points in time (A-F). Scale bar: 10 μm.

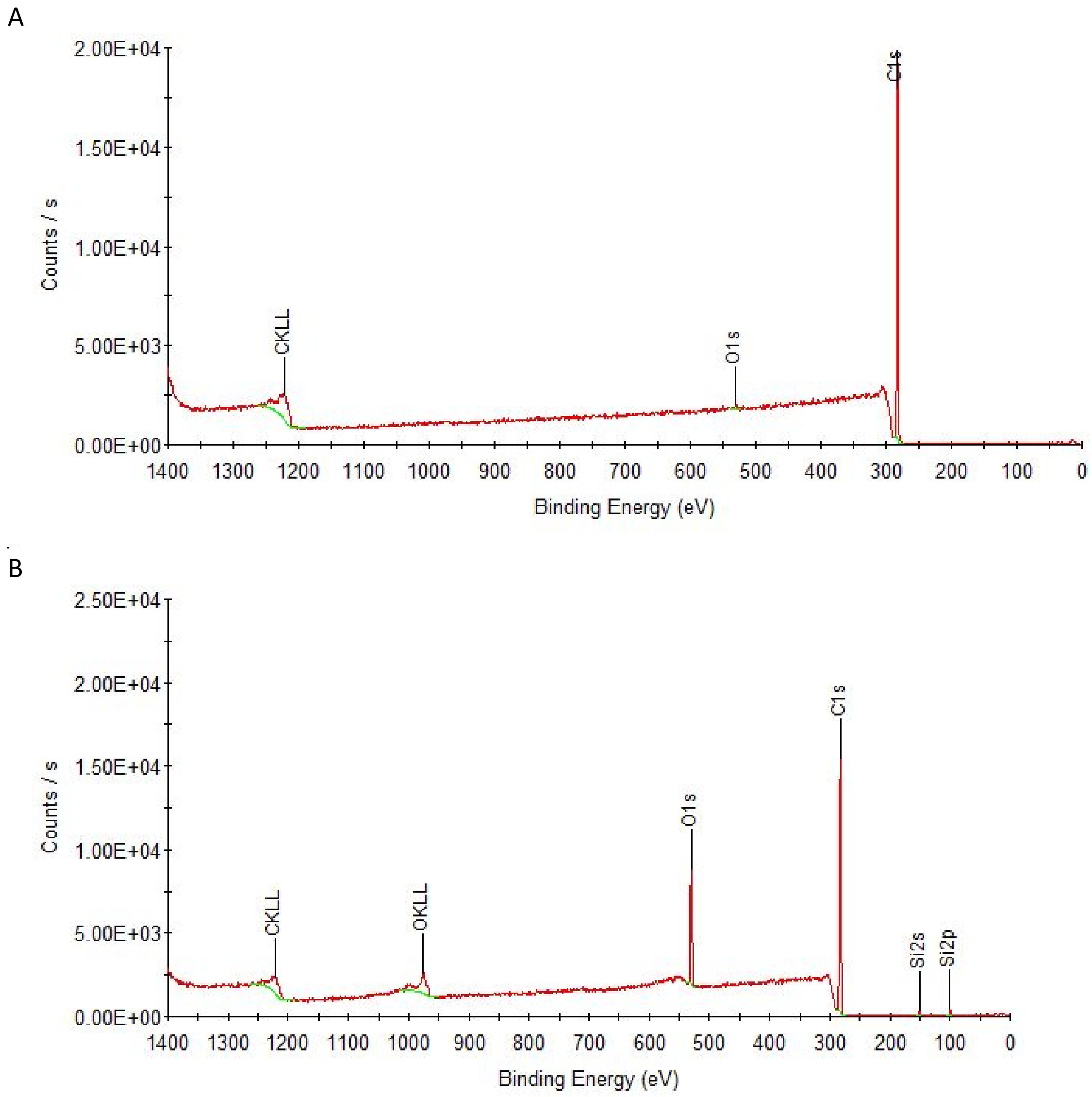

Figure S6. XPS survey spectra of COP before (A) and after (B) GPTS-functionalization.

Table S1. Elemental surface composition of pristine and GPTS-functionalized polymer foils determined by XPS.

Sample	Elemental concentration (at. %)			
	C1s	O1s	N1s	Si2p
COC	99.6	0.4		
COC-GPTS	85.2	13.4		1.4
COP	99.2	0.8		
COP-GPTS	84.7	14.8		1.6
PA6	84.8	7.8	7.4	
PA6-GPTS	73.4	16.5	8.4	1.7
PMMA	77.7	22.3		
PMMA-GPTS	73.4	26.0		0.6

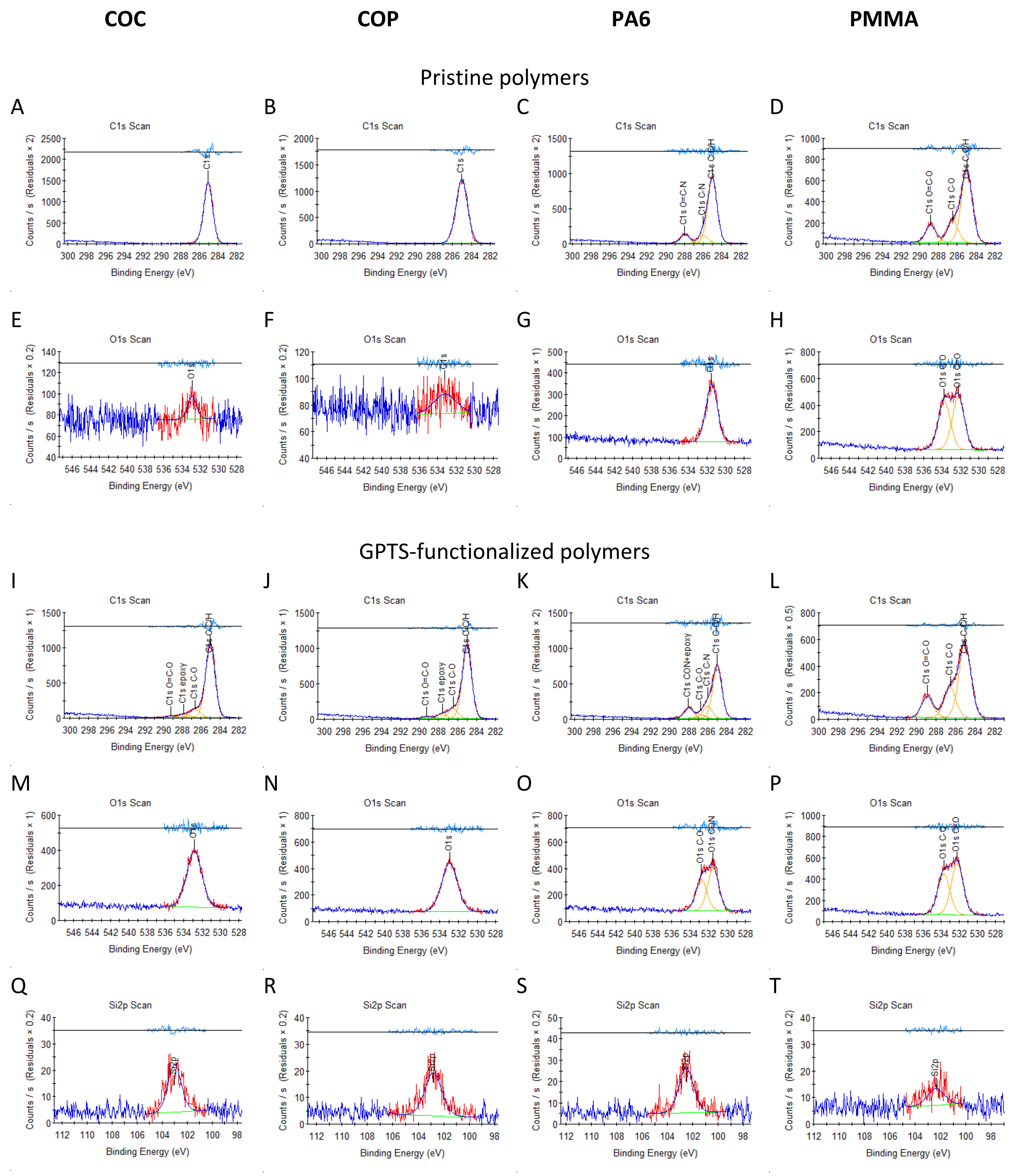

Figure S7. XPS high resolution spectra of COC, COP, PA6 and PMMA polymers before (A-H) and after (I-T) GPTS-functionalization (C1s, O1s and Si2p photoelectron peaks).
